# Distinguishing delusional beliefs from overvalued ideas in Anorexia Nervosa: An exploratory pilot study

**DOI:** 10.1186/s40337-022-00600-2

**Published:** 2022-06-23

**Authors:** Rachel Barton, Phillip Aouad, Phillipa Hay, Geoffrey Buckett, Janice Russell, Margaret Sheridan, Vlasios Brakoulias, Stephen Touyz

**Affiliations:** 1 School of Psychology, Faculty of Science, University of Sydney, Sydney, NSW Australia; 2 InsideOut Institute, Central Clinical School, Faculty of Medicine and Health, University of Sydney, Sydney, NSW Australia; 3 School of Medicine, Western Sydney University, Sydney, NSW Australia; 4 Translational Health Research Institute, Western Sydney University, Sydney, NSW Australia; 5 Eating Disorder Unit - Northside West Clinic (Ramsay Mental Health), Wentworthville, NSW Australia; 6 Royal Prince Alfred Hospital, Sydney, NSW Australia; 7 Northside Clinic (Ramsay Mental Health), St Leonards, NSW Australia; 8 Western Sydney Local Health District Mental Health Service, Sydney, NSW Australia

**Keywords:** Anorexia nervosa, Eating disorders, Delusion, Beliefs, Body image, Dissatisfaction, Perception

## Abstract

**Background:**

Characterised by the belief that more weight needs to be lost—despite emaciation, failing organs, medical instability and prospect of death—Anorexia Nervosa (AN) is a condition in which irrational, and highly-skewed, beliefs can be of delusional intensity. However, the nexus between delusion and rational awareness and how this is related to body image acceptance and perception has yet to be examined in AN. The current study aims to investigate the relationship between body dissatisfaction and beliefs of delusional intensity in an adult AN inpatient sample.

**Methods:**

Twenty-one adults (n_(women)_ = 20; n_(men)_ = 1), with a mean age of 27 years old (SD = 10), presenting for inpatient treatment for AN (ranging in severity from mild to severe; *M*_(Body Mass Index)_ = 17 kg/m^2^; *M*_(Length of Stay)_ = 22 days) participated in the study. Participants’ dominant beliefs (related to AN) and level of insight (delusional; overvalued idea; or fair insight) were measured using either the Brown Assessment of Beliefs Scale (BABS) or the Nepean Beliefs Scale (NBS). The degree of body dissatisfaction was determined by examining the discrepancy between “perceived” and “ideal” body perception. To determine subjective and objective beliefs both the Contour Drawing Rating Scale (subjective) and computerised Body Image Assessment Software (objective) were used.

**Results:**

Almost one quarter (23.7%; *n* = 5) of participants appeared to have beliefs of delusional intensity related to their body shape (*M* = 27.4; *SD* = 23.03). Although a positive linear trend was indicated, there were no significant differences in body dissatisfaction scores between level-of-insight. Individuals whose belief was categorised as delusional were more likely to hold a negative affective body image state based on their ratings on the body image state survey when compared to the group who had good/fair insight (95% CI [0.53, 18.19]; *p* = 0.03).

**Conclusions:**

The current exploratory pilot study concurs with others in the published literature that demonstrate that approximately 25 percent of participants with AN may have delusional ideas. The implications for treatment in similar samples warrant attention. Future research should also seek to understand the clinical significance of this delusional categorisation, the benefits of its utility in this population, and its relation to the severity of AN or stage of illness.

## Plain English Summary

Anorexia Nervosa (AN) is notable for the individual’s conviction that they need to lose weight—even when they are dangerously underweight- or to prevent weight gain. However, how these beliefs relate to the emotional experience of the body, in the presence of AN need to be explored further. This study begins to examine this relationship. Twenty-one adults (95% women; average age 27 years) receiving in-patient treatment for AN were asked a series of questions related to beliefs about their body. To allow a comparison between the level-of-insight and body shape dissatisfaction, participants were also asked to rate how satisfied or dissatisfied they were about their body shape. Almost 25% of the sample had beliefs of delusional intensity about their body shape. Further, it was also found that people with AN who had stronger beliefs of delusional intensity were more likely to be dissatisfied with their body shape. This pilot study appears to support previous research that suggests that some individuals with AN have stronger beliefs of delusional intensity. The findings from the current study should be extended with future research looking at the impact of beliefs on illness and treatment.

## Background

Anorexia Nervosa (AN) is characterised by cognitive and behavioural disturbances, which result in individuals with the illness becoming preoccupied with maintaining a dangerously low weight [1]. The core cognitive features centre on intense idealisation of thinness, fixed beliefs about eating, shape and weight and a central fear of gaining weight [2]. Given that AN is distinguished by a pervasive desire and drive for thinness, a state incessantly maintained even when a deathly emaciated state is reached, people with AN may hold beliefs about their body size, proportions, or weight which may not be consistent with reality and are not amenable to contradictory evidence—such as BMI or visible signs of emaciation [3–5]. Additionally, recent evidence suggests that a subgroup of individuals with AN may hold beliefs of delusional intensity about weight, shape and appearance [6]. However, little is known about the cognitive features that might distinguish this subgroup. Beliefs represent cognitive appraisals, they guide emotion and behaviour, are infrequently questioned and generally accepted as a person’s absolute truth [7]. The person with AN mostly conveys their beliefs in reference to their weight, shape or appearance (through statements such as: “I feel I am too fat”) of their body or body part [8]—with such beliefs often being classified as ‘overvalued ideas’ (OI) [9].

There is a subtle difference between OI’s and beliefs of delusional intensity (commonly known as: delusional beliefs). OI's are beliefs understood by the individuals as truths, they are irrational in nature and held with high conviction but leave room for some element of doubt when challenged by contradictory evidence [9]. In contrast, beliefs that are implausible, fixed and held with high conviction despite competing evidence (without evoking uncertainty) are considered delusional [2].

Several studies have identified a subset of individuals with AN that experience beliefs of delusional intensity (as opposed to the majority of individuals with AN, who are believed to hold OIs) about weight and shape, fear of losing control and body image concerns. The prevalence of beliefs of delusional intensity in these samples ranges from 10–28.2% [6, 10–12]. Beliefs of delusional intensity in AN have frequently been shown to correlate significantly with an intense desire for thinness—as measured by the Eating Disorders Inventory (EDI) [13]. This suggests that irrespective of Body Mass Index (BMI) and illness duration, delusional thinking in AN individuals may be underpinned by an intense drive for thinness [6, 10, 11]. Other cognitive processes that Konstantakopoulos and colleagues [12] found to characterize this delusional AN profile were: 1) overall body dissatisfaction; 2) a restrictive-dieting subtype; and 3) early age of onset of illness. Nonetheless, the differentiation between individuals with AN that hold beliefs of delusional intensity and how these relate to an individual’s own appraisal of their appearance, remains relatively understudied.

### Rationale

Current first line psychological treatment for AN, does not consider the treatment of beliefs of delusional intensity nor is there any research examining their treatment outcomes. Furthermore, the fifth edition of the Diagnostic and Statistical Manual of Mental Disorders (DSM-5) [2] does not adequately allow clinicians to indicate a person’s level of insight, which is often a marker for a delusional presentation, with greater insight likely leading to more gains in treatment [14, 15]. A cited indicator of illness prognosis in AN, is level of insight [16] with poor insight boding negatively for treatment, being related to later access for treatment and prolonged symptomatology [17]. Therefore, insight is likely an important factor and might subsequently moderate the severity of certain cognitive and behavioural components of the disorder.

### The current study

The present exploratory pilot study will evaluate the relationship between beliefs of delusional intensity in people with AN undergoing inpatient treatment using a novel measure of body image disturbances. This will be among one of the first few studies to use two clinical measures of delusionality—specifically, the Brown Assessment of Beliefs Scale (BABS) [18] and the Nepean Beliefs Scale (NBS) [19] with two body dissatisfaction measures – the Contour Drawing Rating Scale (CDRS) [20] and Body Image Assessment Software (BIAS) [21] examining a clinical cohort of individuals with AN for their mental representation of their “perceived” and “ideal” body. Based on findings from previous studies, it is expected that a subgroup of AN participants will present with beliefs that may be defined as delusional [2]. The current study will aim to distinguish whether individuals with beliefs of delusional intensity demonstrate greater dissatisfaction with their body (as measured by larger discrepancies between ratings of “perceived” and “ideal” body size). It is therefore hypothesised that the subgroup with beliefs of delusional intensity will hold a particularly large discrepancy in their self-rated perceived and desired figure, and thus a greater dissatisfaction with their body image.

## Method

### Design

The current study examined the relationship between body image dissatisfaction and beliefs of delusional intensity in an inpatient population diagnosed with AN. The experiment used a between-subjects design: those with AN who *have* beliefs of delusional intensity *vs.* those with AN who *do not* have beliefs of delusional intensity (over-valued idea and good/fair insight). Belief intensity was so determined by scores on two delusional belief questionnaires. The independent variable had two levels: NBS [19] and BABS [18] (both validated semi-structured assessments of delusionality of beliefs). The dependent variables were the delusional beliefs scale scores and body dissatisfaction (discrepancy between the “ideal” and “perceived” body) scores produced by the two measures CDRS [20] and Body BIAS [21]. Participants completed a series of self-report surveys to assess eating disorder symptoms (Eating Disorder Examination Questionnaire, EDEQ [22]; Body Image State Survey, BISS [23]; and Body Shape Questionnaire, BSQ [24]). Ethical approval was granted for the study by the Human Ethics Committee under delegated authority to the University of Sydney Human Research Ethics Committee and the Northside Group Ethics Committee Protocol.

### Participants

Twenty-one adults diagnosed with AN, according to DSM-5 criteria [2], presenting for inpatient treatment were recruited from two private eating disorders inpatient clinics from across Sydney, Northside Clinic Greenwich (*n* = 9) and Northside West Clinic Wentworthville (*n* = 12). Diagnosis was confirmed by the treating medical teams and medical records. Participants were assessed using the EDEQ [22], a self-report measure of eating disorder psychopathology in a preceding 4-weeks. Participants that scored ≥ 2.25 on the EDE-Q Global score were included in the current study, as these were above community norms and in line with clinical sample norms (*M* = 3.64; *SD* = 1.47) [22, 25]. Participants were medically stable as indicated by vital signs and were regularly assessed by medical practitioners at each site and as part of routine clinical care. Participants with known concurrent comorbidities of substance abuse disorder and a history of psychosis were excluded. Written informed consent was obtained from all participants. Characteristics of the sample can be seen in Table 1.

**Table 1:** Sample characteristics: mean (SD) age, Body Mass Index (BMI), weight, frequency of gender, length of stay in the psychiatric unit, severity of illness (based on DSM-5 severity specifiers) and delusional beliefs scale scores

	Mean (SD)
Age (years)	27 (10)
Weight (kg)	48 (8)
BMI (kg/m^2^)	17 (2)
Length of stay (days)	22 (18)
Severity (DSM-5)	Moderate (mild-severe)

	N
Sex	20 Female
	1 Male
Delusional beliefs scales	
Nepean beliefs	9
Browns assessment of beliefs	12

### Procedure and measures

#### Self-report measures

Participants first completed a series of self-report surveys to assess eating disorder symptoms and body dissatisfaction. The surveys were hosted online using the Qualtrics platform and delivered via an application presented on an Apple™ I-pad (EDEQ [22], BISS [23], and BSQ [24]).

*Eating Disorder Examination Questionnaire (EDE-Q)* [22] assesses the frequency of eating disorder cognitions and behaviours over a 28-day period. The 28 item self-report provides a global score from averaging 4 subscales (restraint (5-items), eating concerns (5-items), shape concerns (8-items), and weight concern (5-items)). Internal consistency for the current sample was excellent (α = 0.91) with excellent validity.

*Body Shape Questionnaire (BSQ)* [24] assesses the frequency of weight, shape and appearance concerns over the past 4-weeks on a Likert scale (“Never” 1 – 6 “Always”). Cut off points were used to indicate shape concerns < 80 (no concerns), 80 – 100 (mild concerns), 111 – 140 (moderate concerns) and > 140 (marked concerns). Internal consistency of the current sample was excellent (α = 0.96) and the BSQ has been well validated [24].

*Body Image State Survey (BISS) *[23] is a measures an individual’s affective body-image state. The six 9-point item self-report provides a global score, with body image states being indicated as more positive with higher scores and more negative body image state as lower scores [23]. Internal consistency of the scale was acceptable (α = 0.69) with good validity.

#### Body dissatisfaction measures

Participants’ body dissatisfaction was then measured by examining their mental representation of their “real” and “ideal” body using BIAS [21] a computer administered program and the CDRS [20] a figure rating scale presented via Qualtrics on the Apple™ I-pad.

*Contour Drawing Rating Scale (CDRS)* [20] asked participants to select from nine options of female and male bodies of gradually increasing body sizes (“underweight” 1 – 9 “larger-bodied”) first for their current body size and then for their ideal body size. The discrepancy between the “present” and “ideal” body size represented a measure of body dissatisfaction, where higher scores represent greater body dissatisfaction. Thompson & Gray [20] have demonstrated acceptable test–retest reliability (*r* = 0.78) and concurrent validity (*r* = 0.71).

*A Body Image Assessment Software (BIAS)* [21] was a virtual task installed on a Windows laptop and used with Microsoft Access 2000. The program displayed a frontal and profile (side) view of a female human figure. Participants were asked to modify 6 body parts (head, arms, breast, waist, hips and legs) on the frontal view and 5 body parts (head, breast, waist, hips and legs) on the profile view. Participants interacted with a control panel to increase and decrease in size each of the body parts. Participants altered the figure under two conditions. The first condition asked participants to manipulate the figure to closely reflect their real (perceived) body image at present. The second condition asked participants to manipulate the figure to closely reflect their ideal (desired) body image. The program generates a measurement in twip where 576 twips equates to 1 cm. For the purpose of the current study the data generated was evaluated as an original twip score. The total discrepancy between scores taken from the perceived and desired body represented a body dissatisfaction index. The greater the discrepancy between the perceived and desired body represented greater bodily dissatisfaction. Ferrer-García, & Gutiérrez-Maldonado [26] demonstrated the BIAS has excellent internal consistency (Cronbach’s α = 0.94) and good convergent validity (*r* = 0.69; *p* < 0.001) with a valid body dissatisfaction scale (Body Image Assessment-Revised) [27], suggesting BIAS is a good measure of body dissatisfaction.

#### Delusional belief intensity measures

Beliefs of delusional intensity were assessed using the Browns Assessment of Beliefs Scale (BABS) [18] and the Nepean Beliefs Scale (NBS) [19]. Both the BABS and NBS scales use open questioning to elicit an agreed upon dominant belief associated with the individual’s illness. Following these, closed questions were used to quantify and assess the level of delusional thinking associated on a spectrum of good insight to an overvalued idea to delusional thinking. The reliability of the interviewing technique employed was analysed by comparing 20% of the BABS/NBS interviews with another trained research assistant (RA). The independent scores for beliefs of delusional intensity were examined using Cohen’s kappa and was high for BABS (*k* = 1.0) and NBS (*k* = 1.0) Index Scores.

The BABS [18] is a semi-structured interview that assesses 6 characteristics (conviction, perception of others’ views of beliefs, explanation of differing views, fixity of ideas, attempts to disprove ideas and insight) of beliefs on a 5-point Likert scale (0–4), reaching a total score ranging from 0–24. Cut-off points were used to differentiate insight on a continuum using the following scores “good/fair insight” (0–12), to a belief as an “over-valued” idea [13–17] to a belief being “delusional” (18 +). Internal consistency of the scale was satisfactory (α = 0.67). The BABS has previously been used in AN samples [6].

The NBS [19] also comprises a semi-structured approach that assessed 5 characteristics (conviction, fixity, fluctuation, resistance, and awareness that the belief is unreasonable) of beliefs on a 5-point Likert scale (0–5), reaching a total score ranging from 0–20. Correlational data between the BABS and NBS indicated cut off scores for delusional intensity as 0–10 for “good/fair insight”, 11–15 to a belief as an “over-valued idea” and 16 + to a belief being “delusional”. Internal consistency of the scale was good (α = 0.89).

### Statistical analysis

The study adopted an exploratory approach to analysis. Data were inspected for outliers and normality. A chi-square test of independence was used to assess the association between frequency of beliefs (delusional, overvalued idea and insightful) and belief scale. Comparisons between belief groups (delusional, overvalued idea and fair/good insight) on assessment scores and clinical features (BMI, Severity, Illness duration, length of stay in the inpatient unit, EDEQ global score, BSQ, BISS, CDRS and BIAS) were made using one-way ANOVA’s. A post hoc Tukey’s test followed up significant interactions to determine effects between delusion groups. Pearson’s product moment correlation coefficients were computed to compare the strength of association between total delusional beliefs score and clinical scores (EDEQ global score, BSQ, BISS, CDRS and BIAS). Statistical analyses were performed using IBM SPSS version 24. Two tailed statistical tests were used, and the alpha level was set at 0.05. Given our limited sample size and the numerous comparisons undertaken, the alpha level was not adjusted for multiple comparisons to avoid the chance of a type 1 error. Effect sizes were calculated for group differences as omega-squared ω^2^ (ω^2^ > 0.01 = small effect, ω^2^ > 0.06 = medium effect, ω^2^ > 0.14 = large effect) [28].

## Results

**Fig. 1:**
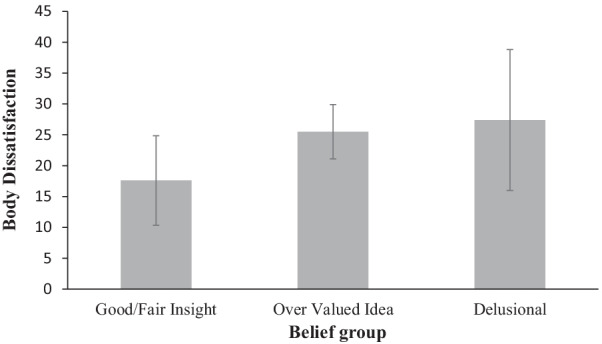
Mean body dissatisfaction score for beliefs assessed as fair insight, overvalued idea and delusional across both the BABS [18] and the NBS [19]

The mean delusional beliefs score (level-of-insight) in the sample for the BABS was 13.7 (*SD* = 4.1) and the NBS was 14.3 (*SD* = 1.7). Across both scales, of the 21 beliefs assessed, 6 (28.6%) participants showed good/fair insight, 10 (7.6%) overvalued ideas and 5 (23.7%) had delusional intensity of beliefs. A chi-square test of independence was conducted between belief group (delusional, overvalued idea and insightful) and belief scale (BABS and NBS). There was no statistically significant association between belief group and belief scale, χ^2^ (2)  = 3.714, *p* = 1.56. Several cells had an expected count less than 5, therefore the assumption of cell count less than five was violated and the result may not be valid and should be interpreted with caution. Across both scales (BABS and NBS) the mean body dissatisfaction score for each belief category was 18.3 (*SD* = 17.6) good/fair insight, 25.5 (*SD* = 13.01) overvalued idea and 27.4 (*SD* = 23.03) delusional belief, where higher scores indicate greater body dissatisfaction. The mean body dissatisfaction scores as a function of belief category collapsed across delusional beliefs scale are presented in Fig. 1.

One-way ANOVAs were used to test whether measures of psychopathology (BMI, length of stay (LoS), severity of illness (SoI), duration of illness (DoI)), body shape (EDEQ, BSQ, BISS) and dissatisfaction (CDRS and BIAS) differed according to belief category (fair insight, overvalued idea and delusional). There were no outliers and data were normally distributed as assessed by Shapiro–Wilk test (*p* > 0.05) for each group. Finally, homogeneity of variance was met as assessed by Levene’s test of homogeneity of variances (*p* > 0.05).

There were no significant differences between participants based on their belief category on measures of psychopathology as well as quantitative measures, namely the EDEQ, BSQ and the two measures of body dissatisfaction (see Table 2).

**Table 2:** Results of one-way ANOVA tests between belief categories and demographic and clinical features of participants

	Mean (*SD)*			*F*	*p*	ω^2^
	Fair Insight	Overvalued	Delusional			
Participants (n)	6	10	5			
BMI (kg/m^2^)	17.12 (3.17)	17.15 (2.75)	17.48 (3.74)	0.02	0.97	− 0.10
DoI (years)	2.66 (2.5)	11.2 (11.37)	13.60 (7.3)	2.50	0.10	0.12
SoI	Moderate	Moderate	Moderate	0.12	0.80	− 0.09
LoS	15.66 (6.31)	19 (14.6)	36.4 (27.91)	2.34	0.12	0.11
EDEQ (global)	4.21 (0.87)	4.28 (1.46)	4.62 (0.8)	0.19	0.82	− 0.08
BSQ	145 (28.31)	144.4 (41.11)	164.2 (30.18)	0.57	0.57	− 0.04
**BISS**	**30.16 (7.6)**	**15.00 (4.66)**	**10.80 (5.06)**	**3.73**	0.04^*^	**0.20**
CDRS (dissatisfaction)	2.83 (2.48)	2.90 (1.91)	5.2 (2.94)	1.88	0.18	0.07
BIAS (dissatisfaction)	18.33 (17.64)	25.5 (13.01)	27.4 (23.03)	0.47	0.63	− 0.05

Correlational data between the BABS [18] and the NBS [19] was used to determine delusional intensity category score ranges: good/fair insight (0–10); overvalued idea (11–15); delusional belief (16 +). *Abbreviations****:*** DoI = Illness Duration (years); SoI = Severity of Illness (based on DSM-5 severity specifier); LoS = Length of Stay (days); EDEQ = Eating Disorder Examination Questionnaire (Global Score); BSQ = Body Shape Questionnaire; BISS = Body Image State Survey; CDRS = Contour Drawing Rating Scale (dissatisfaction subscale); BAIS = Body Image Assessment Software (dissatisfaction subscale). *Significant *p* < 0.05

Participants differed significantly on scores on the Body Image State Survey across belief category, *F*(2, 18)  = 3.73, *p* = 0.04, ω^2^ = 0.2. Body Image State scores decreased in order from Fair insight (*M* = 30.16, *SD* = 7.60) to over-valued idea (*M* = 15.00, *SD* = 4.66) and beliefs of delusional intensity (*M* = 10.80, *SD* = 5.06), where more negative body image states were indicated by lower scores. Tukey post hoc analysis indicated that the difference in BISS scores (9.36, 95% CI [0.53, 18.19]) showed a significantly lower mean score in the delusional group than the fair-insight group (*p* = 0.03). No other group differences were statistically significant. Effect sizes (omega-squared, ω^2^) for the differences between belief categories across measures, ranged from small to large. Due to the small sample size, results should be interpreted with caution, as we may not have had sufficient power to detect effect if present.

A Pearson’s product-moment correlation (*r*) was used to assess the relationship between scores of delusional intensities on the BABS and NBS with measures of psychopathology (BMI, length of stay, illness severity, illness duration), body shape (EDEQ, BSQ, BISS) and dissatisfaction (CDRS and BIAS), as shown in Table 3. A Shapiro-Wilks test indicated that relationships between variables were linear and normally distributed (*p* > 0.05), with no outliers. Both BABS (*r* = − 0.63) and NBS (*r* = − 0.78) total scores were significantly negatively correlated with the Body Image State Survey (*p* = 0.02). There were no other statistically significant correlations.

**Table 3:** Correlations between clinical features and the Brown Assessment of Beliefs Scale [18] and the Nepean Beliefs Scale [19] total scores

	BABS (*r*)	*p*	NBS (*r*)	*p*
EDEQ	0.29	0.36	0.48	0.18
BSQ	0.27	0.38	0.58	0.09
BISS	− **0.63**	0.02^*^	− **0.78**	0.02^*^
CDRS (dissatisfaction)	0.29	0.36	0.54	0.12
BIAS (dissatisfaction)	0.33	0.28	0.46	0.21

Pearson’s correlation *(r)* coefficients were used to determine associated belief category cut-off scores between the BABS [18] and the NBS [19]. *Abbreviations****:*** EBABS = Brown Assessment of Beliefs Scale (total score); NBS = Nepean Beliefs Scale (total score); EDEQ = Eating Disorder Examination Questionnaire (Global Score); BSQ = Body Shape Questionnaire; BISS = Body Image State Survey; CDRS = Contour Drawing Rating Scale (dissatisfaction subscale); BAIS = Body Image Assessment Software (dissatisfaction subscale). *Significant *p* < 0.05

The content of participant’s self-reported beliefs were also examined for any consistent thematic relationships. Upon observation, several themes related to a fear of gaining weight, control-seeking, self-worth, negative evaluation and other categories were extracted from the sample. Overall, the percentage of participants that endorsed the five main themes were 6 (28.5%) showed a fear of gaining weight, 2 (9.5%) showed control-seeking beliefs, 7 (33.3%) related to self-worth, 4 (19.1%) related to negative evaluation and 2 (9.5%) reported other types of beliefs. The number of participant’s beliefs associated with each theme and examples, can be seen in Table 4.

**Table 4:** Emerging themes observed in participants beliefs

Themes	Belief example	n (%)
Fear of weight gain	“If I gain weight I will be disgusting”	6 (28.5%)
Control Seeking	“Putting on weight means I will lose control in my life”	2 (9.5%)
Self-worth	“I am worthless as a person because my body is disgusting”	7 (33.3%)
Negative evaluation	“I will get fat and be ugly, undesirable, ignored and ridiculed if I eat 3 meals a day”	4 (19.1%)
Other	“I don't deserve to have food”	2 (9.5%)

## Discussion

The present exploratory pilot study investigated the relationship between body dissatisfaction and beliefs of delusional intensity in an inpatient population with AN. Our results indicated that 23.7% (n = 5) of the sample study reported a belief about their core symptomatology that could be classified of delusional intensity according to the NBS and BABS delusional belief rating scales. Of the 21 subjects, 20 reported body dissatisfaction as indicated by larger overall representations of their perceived body when compared to their ideal. Despite there being a positive linear trend between body dissatisfaction and beliefs of delusional intensity, there were no significant differences in body dissatisfaction scores between belief categories (delusional, overvalued idea and fair insight). Individuals whose belief was categorised as delusional were more likely to hold a negative affective body image state based on their ratings on the body image state survey, but only when compared to the group who had fair insight into their belief. There were no other differences between groups on measures of illness severity and psychopathology. Anecdotally, beliefs with themes relating to the negative evaluation of others were rated as being delusional more than any other theme.

The identification of a subgroup of participants with beliefs of delusional intensity is in line with previous findings. In our sample the proportion of cases presenting with a beliefs of delusional intensity (23.7%, N = 21) was more than Steinglass and colleagues [6], Hartmann and colleagues [11], and Mountjoy and colleagues [10] (20%, N = 20; 16%, N = 19; and 10%, N = 20 respectively) despite retaining similar sample sizes. This might have been due to participants in our study being entirely made up of an inpatient population with AN, whilst prior studies incorporated a mixture of in- and outpatient populations with AN. It is possible that inpatient populations of people with AN may be more likely to hold and express beliefs of delusional intensity, and that the presence of beliefs of delusional intensity might have directly contributed to their inpatient status. Despite these minor discrepancies, previous research and current results indicated that beliefs concerning the psychopathology of AN can reach delusional proportions in this population. Our findings support recommendations by authors for the inclusion of insight specifiers of fair insight, overvalued idea and delusional in current diagnostic classifications systems for AN.

Our findings do not, however, confirm the relationship between body dissatisfaction and beliefs of delusional intensity in AN therefore our hypothesis was not supported. This appears to be in line with Hartmann and colleagues [11], who found no relationship between beliefs of delusional intensity and two self-report scales used to assess general body image disturbances. Our results further support Hartmann and colleagues [11], conclusion that beliefs of delusional intensity may not be associated with the cognitive and evaluative component of body image. We did find a relationship between a negative body image state and delusionality, which was consistent across both the BABS and NBS. These findings appear to be in line with Konstantakopoulos and colleagues [12], who found delusional psychopathology to be positively correlated with an Eating Disorder Inventory (EDI) [13] subscale measuring discontentment with the body. Therefore, our findings appear to support both Konstantakopoulos and colleagues [12] and Hartmann and colleagues [11], conclusions that delusional beliefs in individuals with AN may be associated with the affective state held toward the body rather than the perceptual component. Alternatively, given the small clinical sample, it might be that our study lacked sufficient power to detect a relationship between delusionality and body image dissatisfaction even if it were present. Indeed, a suggestive trend was observed in our data, in the hypothesised direction. Future research should re-examine this relationship with a larger sample capable of finding a small to medium sized effect.

The implications for treatments and subsequent prognosis for individuals presenting with fixed unshakeable beliefs about weight, shape and appearance are not yet clear. Although not significant, there was some indication of a relationship between beliefs of delusional intensity and body image dissatisfaction. Given that there is a population presenting with such delusional intensity beliefs, some adjustments to cognitive-behavioural components of treatment might be clinically relevant based on recommendations outlined in Cognitive Behaviour Therapy for Psychosis [29]. These individuals might be particularly sensitive to having their highly regarded belief challenged, especially in the absence of a strong therapeutic relationship. For example, therapists might address the person’s beliefs of delusional intensity with more openness, hesitancy and curiosity than an overvalued idea. Instead of using a strength of belief rating, asking individuals to rate their associated distress and preoccupation might be better received. Using motivational interviewing techniques to ascertain the helpful/unhelpful and positive/negative aspects of their beliefs, may also facilitate flexibility, insight and motivation toward change in this population.

With regard to ideas of delusional intensity, the therapeutic efficacy of pharmaceutical interventions for AN, specifically the role of antipsychotic agents such as olanzapine, in the reduction of delusional beliefs in AN has yet to be conclusively established [30–35]. Clinical experience, however, would suggest that this medication is of some value at least in reducing the associated distress.

### Limitations

The current study had several limitations. Firstly, it is evident that type II errors have not been adequately controlled for given the small sample size. A post-hoc analysis (G*Power) [36] indicated that large effect sizes (*d* = 0.94) had to be assumed in order to detect between group differences, which required a minimum sample size of 66 participants. Secondly, both the NBS and BABS require further validation in AN samples. Specially, to our knowledge the NBS has not been used with an eating disorders population; and further validation for the use of the BABS in larger AN samples is required [6]. Thirdly, the quality of participant information, particularly in reference to their clinical history and current diagnosis was limited by medical history notes which failed to account for known historical and current comorbidities as well as specific information regarding AN diagnosis (Restrictive or Binge/Purge). This meant some potential clinical characteristics (e.g., comorbidities) may not have been adequately controlled for. Similarly, given the current study examined AN in adults, effect of previous treatments and their impact on the current level of delusion should be controlled for in future studies. Therefore, future studies will need to account and control for this. Finally, all participants were recruited from a “voluntary” inpatient environment which implicates insight and help-seeking behaviours. Despite two measures of delusional beliefs (NBS and BABS) correlating with other clinical measures of AN, the recruited sample was of individuals seeking inpatient treatment and may not be representative of the wider population from which the sample was drawn; thus, findings should be considered preliminary. To that point, given there was an absence of a community-based sample that might evade health-services and potentially present with less insight and more delusional features, the AN population more broadly may not have been adequately represented. Based on the limitations with this sample and procedure, results presented in this study should be interpreted with some degree of caution. The study however had some strengths, it was the first to study the utility of two delusional belief scales (which demonstrated good inter-rater reliability), as well as use two representational measures of body dissatisfaction.

### Future research

Future research in this area is necessary. Firstly, a scale that adequately supports interviewers with eliciting and isolating a core belief is warranted. At present, scales fail to delineate the clinical process of identifying a belief, which is a complex and sensitive process within clinical practice. Secondly, the prognosis and illness trajectory of those with AN who hold beliefs of delusional intensity is unclear—research examining the treatment outcomes and stability of their beliefs is required.

### Clinical significance

Ultimately, the clinical utility of identifying this subgroup of AN individuals with potentially delusional beliefs should be further ascertained in future studies. Specifically, given that more-and-more studies are bringing to light the association between AN and beliefs of delusional intensity, and given that current treatment regimens incorporate antipsychotic pharmacological interventions, links between treatment choices and further justification for such choices needs to be explored further. For example, beyond the broad rationale of serotonergic pathway regulation [37], (how and why) does olanzapine -albeit in lower doses than those necessary to treat psychosis- help to target delusional beliefs in those with AN, if AN is not a psychotic disorder? Particularly, when a majority of studies examining the use of Olanzapine in AN are case studies, and approval by the United States of America Food and Drug Administration (FDA) has not been granted for Olanzapine use specifically in AN, yet it is still widely prescribed in the absence of efficacy and safety evidence [37], with no clear significant benefits on psychological symptoms and only modest significant impact on weight [38].

Further, psychological treatments might be adapted so as to include modules designed to better target delusional belief presentations—as has been successfully done in other psychotic conditions [39, 40]. Overall, the findings of the current study add to a very small area of research, which may potentially provide useful direction and insight into helping treat AN through a potentially different lens to current.

## Conclusion

In Conclusion, AN is one of the most chronic, severe and debilitating psychiatric illnesses to afflict young people, and its prevalence has doubled in the last decade alone [41]. New approaches to reducing morbidity associated with AN are urgently needed. A neglected clinical target relates to beliefs of delusional intensity, which appears to be present in a subgroup of individuals with AN, and which might be associated with more severe aspects of their symptomatology. Studying clinical correlates of beliefs of delusional intensity in AN could help identify whether this group is at elevated risk, and if so, could lead to the development of clinical screening tools, and novel therapeutic approaches for the treatment of AN.

## Data Availability

The datasets generated and/or analysed during the current study may be available upon request. Data requests are at the discretion of the data owner and/or participating institutions.

## Abbreviations

BABS: Brown Assessment of Beliefs Scale
BDD: Body Dysmorphic Disorder
BIAS: Body Image Assessment Software
BISS: Body Image State Survey
BSQ: Body Shape Questionnaire
CBT: Cognitive Behavioural Therapy
CDRS: Contour Drawing Rating Scale
DB: Delusional Belief (aka ‘beliefs of delusional intensity’)
DoI: Duration of Illness (years)
DSM: Diagnostic and Statistics Manual
EDEQ: Eating Disorder Examination Questionnaire
EDI: Eating Disorder Inventory
LoS: Length of Stay (days) in psychiatric unit
NBS: Nepean Beliefs Scale
OCD: Obsessive Compulsive Disorder
OI: Overvalued Idea
RA: Research Assistant
SoI: Severity of Illness-based on DSM-5 severity specifiers
TWIP: Twentieth of a Point (1 twip = 17.64 μm)
